# Bioactive Potential of Marine Macroalgae from the Central Red Sea (Saudi Arabia) Assessed by High-Throughput Imaging-Based Phenotypic Profiling

**DOI:** 10.3390/md15030080

**Published:** 2017-03-20

**Authors:** Stephan Kremb, Constanze Müller, Philippe Schmitt-Kopplin, Christian R. Voolstra

**Affiliations:** 1 Red Sea Research Center, Division of Biological and Environmental Science and Engineering (BESE), King Abdullah University of Science and Technology (KAUST), 23955-6900 Thuwal, Saudi Arabia; sk6225@nyu.edu; 2 Research Unit Analytical Biogeochemistry, Helmholtz Zentrum München, Ingolstaedter Landstrasse 1, D-85764 Neuherberg, Germany; constanze.mueller@helmholtz-muenchen.de (C.M.); schmitt-kopplin@helmholtz-muenchen.de (P.S.-K.); 3 Chair of Analytical Food Chemistry, Technische Universität München (TUM), 85354 Freising-Weihenstephan, Germany

**Keywords:** bioprospecting, natural products, algae, cytological profiling, High-Content Screening, HIV

## Abstract

Marine algae represent an important source of novel natural products. While their bioactive potential has been studied to some extent, limited information is available on marine algae from the Red Sea. This study aimed at the broad discovery of new bioactivities from a collection of twelve macroalgal species from the Central Red Sea. We used imaging-based High-Content Screening (HCS) with a diverse spectrum of cellular markers for detailed cytological profiling of fractionated algal extracts. The cytological profiles for 3 out of 60 algal fractions clustered closely to reference inhibitors and showed strong inhibitory activities on the HIV-1 reverse transcriptase in a single-enzyme biochemical assay, validating the suggested biological target. Subsequent chemical profiling of the active fractions of two brown algal species by ultra-high resolution mass spectrometry (FT-ICR-MS) revealed possible candidate molecules. A database query of these molecules led us to groups of compounds with structural similarities, which are suggested to be responsible for the observed activity. Our work demonstrates the versatility and power of cytological profiling for the bioprospecting of unknown biological resources and highlights Red Sea algae as a source of bioactives that may serve as a starting point for further studies.

## 1. Introduction

Marine organisms have proven to be a rich source of novel and promising bioactive molecules for a wide range of applications, including new therapeutics, cosmetics, and biotechnology [1,2,3,4]. Although marine invertebrates have been the main focus in the search for new marine natural products [5,6], marine algae in particular contribute to the wealth of novel bioactives [7,8,9]. Marine algae have been shown to harbor a plethora of activities, including anti-neoplastic, anti-viral, anti-biotic, anti-oxidant, and anti-inflammatory effects, and in addition have multiple applications in nutrition and cosmetics [3,7,10,11,12]. Natural products in general are a major source of compounds for the treatment of cancer with more than 75% of anti-cancer drugs in clinical trials being either derived or at least inspired by nature. In this respect, marine algae play a special role as they are an increasingly important dietary constituent in large parts of the world and are discussed as potential medicinal foods in the management of cancer [11].

Besides the anti-cancer activity, marine algae show a multitude of anti-viral activities with a substantial number of studies focusing on the human immunodeficiency virus 1 (HIV-1), reflecting the importance of this viral pathogen. HIV-1 continues to be a major human health issue with more than 35.3 million infected individuals worldwide and 2.3 million new infections every year [13]. Algal compounds were found to target different steps of HIV-1 replication, including viral entry [8,14,15] and the major viral enzymes Reverse Transcriptase (RT), Integrase, and Protease [16,17,18]. Moreover, epidemiological findings suggest a correlation of the consumption of marine algae with a low prevalence of HIV/AIDS in parts of Eastern Asia [19,20].

The Red Sea is a largely untapped, uncharted source of bioactives whose waters have not been extensively sampled. The Red Sea extends nearly 2000 kilometers and the semi isolation together with a high salinity and high water temperatures have given rise to unique ecosystems and evolutionary adaptations [21]. Only a few marine algae have been reported from the Red Sea so far (27 entries in AlgaeBase as opposed to 307 for the Arabian Gulf and 512 for the Caribbean) and studies investigating the bioactivity of Red Sea algae are rare. In Red Sea coral reefs, marine macroalgae face several challenges, including competition for space, high solar radiation, high salinity, as well as high water temperatures [22,23]. It is likely that algae as well as other marine organisms have evolved adaptations to tolerate these harsh conditions giving rise to a potential wealth of new bioactives.

Traditionally, the search for bioactivity from natural sources primarily involves target-driven approaches using single-target biochemical assays for primary screening [24,25,26]. While these assays offer high throughput capabilities, their in vitro setting often results in the selection of candidates with poor performance under in vivo conditions or failure due to toxicity issues. In contrast, there are a number of drugs on the market that were initially discovered by involving phenotypic information of the pharmacology on whole organs, animals, or even humans [27]. Given the success of these drugs, it has been proposed that a partial return to phenotypic approaches empowered by modern screening technologies is a worthwhile consideration [27]. To this end, imaging-based High-Content Screening (HCS) has emerged as a promising tool for primary screening of natural products, which produces a wealth of information using both single molecules as well as fractionated biological extracts [28,29,30,31,32]. The outcomes of this technology are more information-rich compared to (traditional) uni-dimensional testing, such as toxicity testing on cancer cell lines as a measure of identifying anti-neoplastic activity. HCS provides information on multiple levels of cell physiology and is able to elucidate mechanisms of compound toxicity [31,32,33,34,35]. Moreover, HCS has been demonstrated to predict compound-related mechanism-of-actions (MOAs) by comparing cellular phenotypes (characterized by cytological profiles) to collections of molecules with known effects on multiple target classes. In addition, cytological profiling is well-suited to elucidate and discover novel and previously unrecognized mechanisms of compound activity.

In this study, we were interested in assessing the bioactive potential of macroalgae from the Central Red Sea. We applied cytological profiling using a highly tailored HCS platform [33] as a method of bioprospecting for novel activities on fractionated extracts of 12 macroalgal species. We show that the highly detailed information resulting from the HCS approach can assist in the selection of promising candidates for further studies and serve as a starting point for chemical analyses, target prediction, and mechanistic studies. In terms of the chemical analysis, we applied a non-targeted strategy. By the implementation of 12 Tesla FT-ICR-MS (Ion cyclotron resonance Fourier transform mass spectrometry), we were able to acquire ultrahigh resolution mass spectra, which describe the overall chemical composition of the algal fractions. Sophisticated data elaboration and filtering led us to groups of structurally similar compounds, which we suggest are responsible for the observed activity.

## 2. Results

### 2.1. Cytological Profiling and Clustering of a Pre-Fractionated Algal Extracts Library

In order to gain detailed insight into the biological activities of algal extracts on human cells, we employed an imaging-based high-resolution phenotypic profiling approach [33]. HCS was used for the profiling of organic extract fractions of twelve macroalgal species from the Central Red Sea (Table 1).

**Figure 1:**
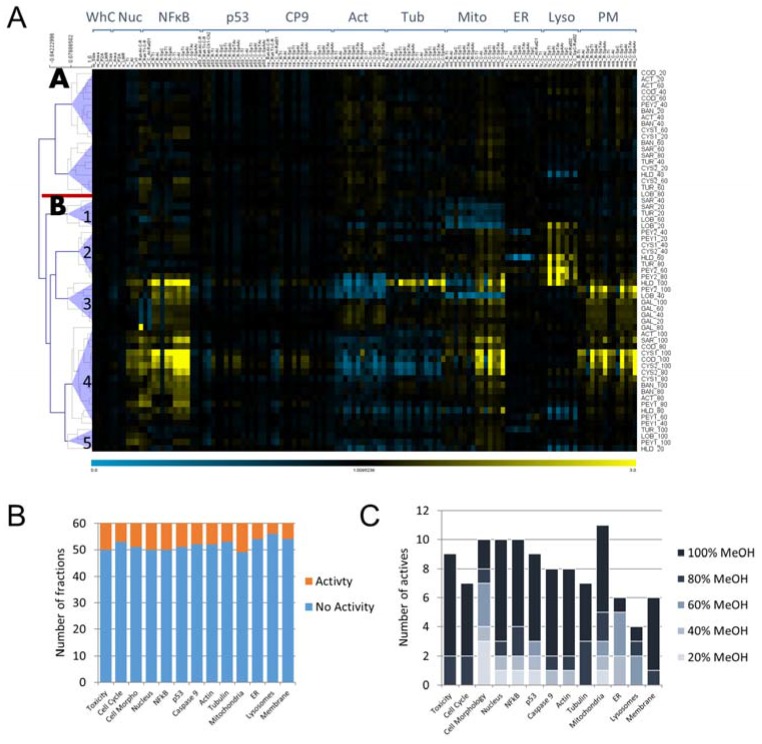
Cluster analysis of cytological profiles from the algal fractions and relative involvement of the cellular markers tested and chemical solvents used. (**A**) Cluster analysis of all cytological profiles of algal fractions (for sample codes see Table 1, attached numbers indicate the percentage of methanol used for elution in solid-phase extraction (SPE)). Colors indicate positive (yellow) or negative (blue) deviation from the mean of untreated control cells for each cellular feature (control = 1). Abbreviations are WhC: whole-cell morphology, Nuc: nucleus, Cp9: caspase 9, tub: tubulin, Mito: mitochondria, ER: endoplasmic reticulum, Lyso: lysosomes, PM: plasma membrane. Pearson correlation was used as a distance metric. The dendrogram depicts distances between individual cytological profiles. Cluster analysis yielded two major clusters and several sub-clusters. The red bar illustrates the separation between the two major clusters and numbers indicate the numbering of sub-clusters in cluster 2; (**B**) Bar chart representing the relative involvement of each cellular marker in the whole set of fractions. A cellular marker was considered as showing activity if at least one cellular feature was exceeding or falling below a certain threshold (Toxicity: below 70% remaining cells; Cell Cycle: ±1× standard deviation; all other cellular markers: ±2× standard deviations); (**C**) Bar chart indicating the relative involvement of the chemical solvent in the yield of positives of the whole set of fractions and all cellular markers. A cellular marker was considered as contributing if at least one cellular feature was exceeding or fell below a defined threshold (see (B)).

Briefly, the used HCS platform employs a combination of 14 cellular markers that inform on key components of cell physiology to generate cytological profiles, which serve as unique fingerprints for compound-induced perturbations. Hierarchical clustering of cytological profiles reveals a clear distinction of two main clusters, subsequently referred to as A and B (Figure 1A). Cluster A combines profiles that show no or only weak effects on the whole panel of cellular markers, whereas cluster B is comprised of cytological profiles that show stronger deviations from the mean for at least one cellular marker and divides into five sub-clusters, B1–B5. Sub-cluster B1 includes fractions of three common brown macroalgae, *Sargassum ilicifolium* (SAR), *Turbinaria turbinate* (TUR), and *Lobophora variegata* (LOB) and is characterized by strong effects on mitochondrial features. Sub-cluster B2 contains fractions with moderate effects on mitochondrial features and strong effects on lysosomal parameters. In contrast, sub-cluster B3 groups profiles that show strong effects on almost all cellular markers except endoplasmic reticulum (ER) and lysosomal features. This sub-cluster encompasses almost all of the fractions derived from *Galaxaura rugosa* (GAL). Sub-cluster B4 is a large group of cytological profiles that resemble profiles in subcluster B3, but are characterized by partly stronger effects on tubulin, mitochondria, and plasma membrane (PM)-related features. This sub-cluster contains almost exclusively fractions that were eluted with high concentrations of methanol (80% and 100%). Finally, sub-cluster B5 resembles sub-cluster B4 with absent effects on the plasma membrane and overall weaker effects.

To compare the overall effects on all cytological markers, we assessed the number of active fractions on each individual marker. Fractions were considered as active on a given marker if at least one of the cytological features exceeded a defined threshold (see Materials and Methods). Activity was uniformly distributed over all cytological markers with a range of 10%–20%. The lowest numbers were found for lysosomal and ER markers as well as for the plasma membrane (Figure 1B). Furthermore, the effect of the chemical eluent used for fractionation by solid-phase extraction (SPE) on the number of actives was analyzed. For the majority of cytological markers, most actives were found in the 100% methanol fraction. In comparison, toxic fractions and those that interfere with the cell cycle, the cytoskeleton, caspase 9, and the plasma membrane were almost all found at high methanol concentrations. Notably, a considerable number of active fractions were also found at lower methanol concentrations particularly for cell morphology, mitochondria, and p53 (Figure 1C).

### 2.2. Cell Cycle Analysis

**Figure 2:**
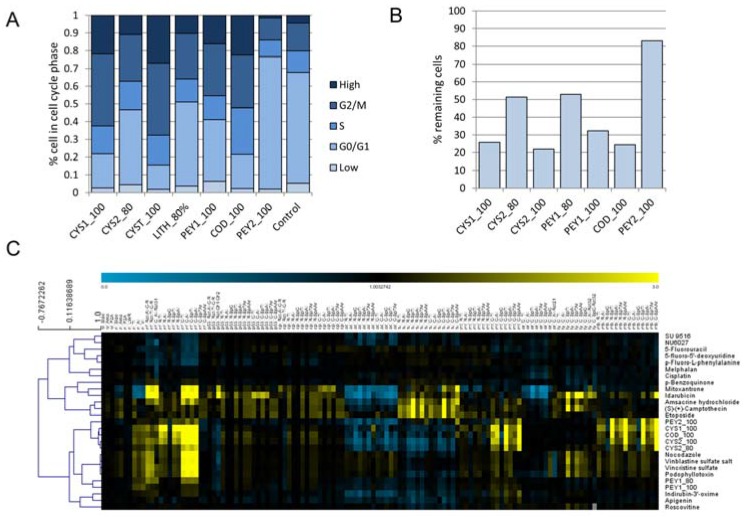
Cell cycle analysis and cytotoxicity of selected algal fractions and underlying cytological profiles. (**A**) Bar chart showing the percentage of cells in each phase of the cell cycle; (**B**) Bar chart showing the percentage of remaining cells in treated wells; (**C**) Cluster analysis of cytological profiles of algal fractions that show an effect on the cell cycle and reference compounds known to interfere with the cell cycle. Colors indicate positive (yellow) or negative (blue) deviation from the mean of untreated control cells (control = 1). For clustering (Pearson correlation) was used.

Several of the algal fractions showed distinct effects on the cell cycle (Figure 2A). All of these were eluted with high methanol concentrations and the extent of the cell cycle arrest correlates with cell loss (Figure 2B). It should be noted that HCS explicitly allows the exclusion of dead or heavily damaged cells from analysis. Thus, the analysis of the cell cycle and cytological profiles is focused on the living cells in the culture and is not an artifact from cell injury or dead cells.

The 100% methanol-fractions from the two *Cystoseira* species show a strong arrest in the G2/M phase together with a significant cell loss. Whereas cell cycle interference by *Cystoseira foeniculacea* (CYS1) was solely detected in the 100% methanol fraction, *Cystoseira myrica* (CYS2) shows a slightly weaker effect in both the 80% and 100% methanol fractions. Two other algal species, *Peyssonnelia squamaria* (PEY1) and *Codium fragile* (COD), showed a significant effect on the cell cycle with a strong arrest in G2/M phase and severe cell loss. Interestingly, COD caused an additional arrest in the S-phase. The cell cycle inhibitory activity of *Peyssonnelia capensis* (PEY2) was different with a moderate arrest in G0/G1 and only weak cell loss.

In order to gain a better understanding of the underlying mechanisms of the cell cycle-related effects, we included profiles of reference compounds with known MoAs from various classes of cell cycle inhibitors (Figure 2C). Pearson clustering showed a good separation into distinct clusters that reflect different functional classes, including inhibitors of tubulin polymerization (TPI: nocodazole, vincristin, podophyllotoxin), cyclin-dependent kinases (CDK: roscovitin, indirubin, apigenin), topoisomerases (amsacrine, p-benzochinone, etopside), DNA synthesis (mitoxantrone, idarubicin), and DNA crosslinking (cisplatin, melphalan). Within these clusters, almost all of the cell cycle-active fractions matched closely to the cytological profiles of TPIs as well as to compounds that are acting on CDKs. Notably, most of the fractions that were found to block the G2/M phase clustered closely to TPIs. The fraction of PEY2 that exhibited an arrest in G0/G1 appears to be more distant from this group. Another exception was represented by the fractions of PEY1. These cytological profiles were clearly distant from the other fractions and found in between the profiles of TPI and CDK inhibitors.

### 2.3. Chemical Analysis of Cell Cycle Interfering Fractions

**Figure 3:**
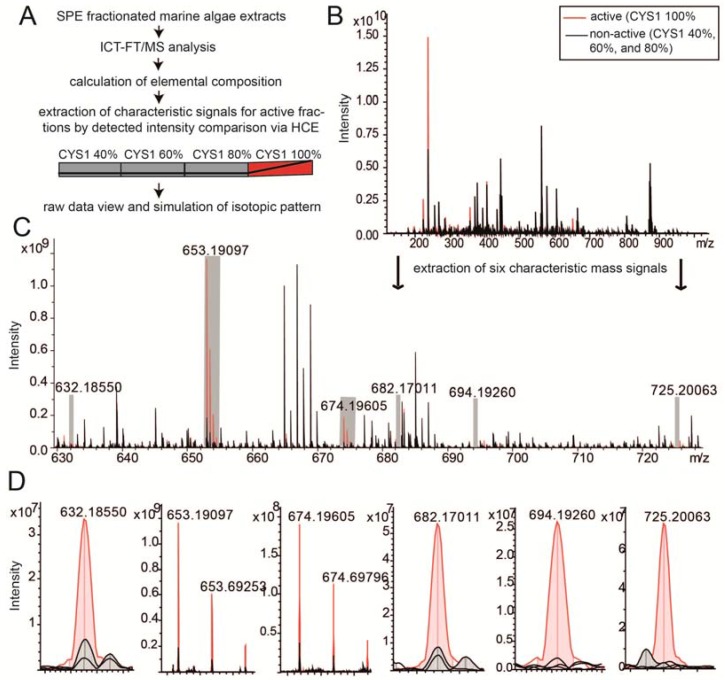
Chemical analysis of the cell cycle interfering fraction of CYS1 (**A**) Overview of the chemical analysis (**B**) Ultra-high resolution mass spectra showing the overlaid data of active (red) and non-active (black) fractions in broad band detection mode (*m*/*z* 122–1300). In each nominal mass 5–10 signals were detected and approximately 2500 formulas were assigned; (**C**) By intensity profiling extracted masses characteristic for the active fraction are in the mass range of 630–730. Each of the mass peaks is highlighted with a gray bar and enlarged for visualization in (**D**).

SPE-fractions of CYS1 were analyzed for their chemical composition via ultra-high resolution MS (Figure 3A). It appears that the composition in each fraction was very complex (Figure 3B); for approximately 2500 signals in each fraction, the elemental compositions could be calculated and were thus included in following data elaboration procedures. Van Krevelen diagrams, which helped to retrieve information from the assigned molecular formulae and enabled graphical differentiation and separation of compound classes displayed these masses [36]. The characteristic and exclusive mass signals in the cell cycle-active fraction were filtered by hierarchical cluster analysis. Six masses appear to be highly present in the active fraction with signal-to-noise (S/N) values above 67 and detected intensities of >2.0 × 10^7^ (Figure 3C,D). Their elemental compositions did not match with previously described natural products. However, the mass defects suggested a complex chemical composition including hydrogen, carbon, oxygen, nitrogen, sulfur, and phosphorous. All of these signals were detected as double charge sodium adducts in the mass range of 630–725. Two of them were also detected as single charge sodium adducts (*m*/*z* 1241.386; *m*/*z* 1283.390), since the original mass spectrum was obtained up to a mass of 1300. The detection of double charge ions suggests an abundance of several functional groups within one structure.

### 2.4. HIV-1 Reverse Transcriptase Activity

**Figure 4:**
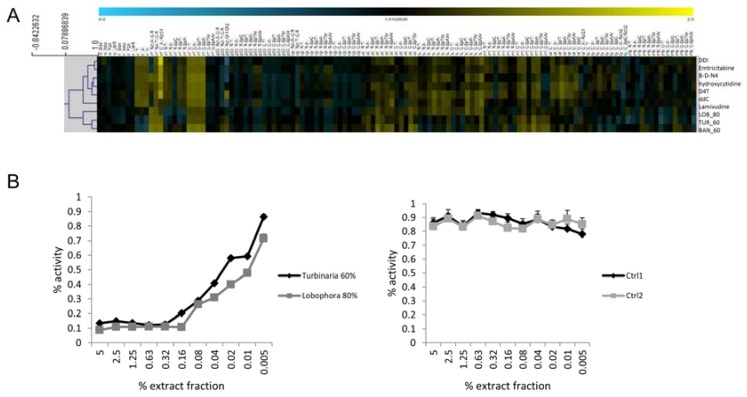
Prediction and validation of HIV-1 reverse transcriptase (RT) inhibitors in selected algal fractions. (**A**) Cluster analysis of cytological profiles from algal fractions and a panel of nucleosidic reference inhibitors of viral proteases. Colors indicate positive (yellow) or negative (blue) deviation from the mean of untreated control cells (control = 1). For clustering, Pearson correlation was used. (**B**) Validation of HIV-1 RT as a biological target for the selected algal fractions. Both fractions show potent dose-dependent inhibition of HIV-1 RT activity in a single enzyme-based assay. Non-matching fractions were used as controls and do not show activity.

We were interested to see if cytological profiling is capable of detecting potential inhibitory activities on a viral enzyme, HIV-1 RT, and thus included a panel of six nucleosidic inhibitors of HIV-1 RT (NRTIs) and other viral polymerases in the analysis (Figure 4A).

All cytological profiles of the six reference RT inhibitors were found to cluster closely with each other. Three cytological profiles of algal fractions matched closely to these reference inhibitors. All of these fractions were derived from brown algae, i.e., TUR, LOB, and CYS2, and were eluted by intermediate (60%) or higher (80%) methanol concentrations. In order to validate the predicted target, the pre-selected fractions were tested for activity in a HIV-1 RT biochemical assay and were found to have a potent inhibitory effect, whereas no inhibitory effect was found by using algal fractions that did not match to the reference HIV-1 RT inhibitors in the cytological profile test (negative control) (Figure 4B).

**Figure 5:**
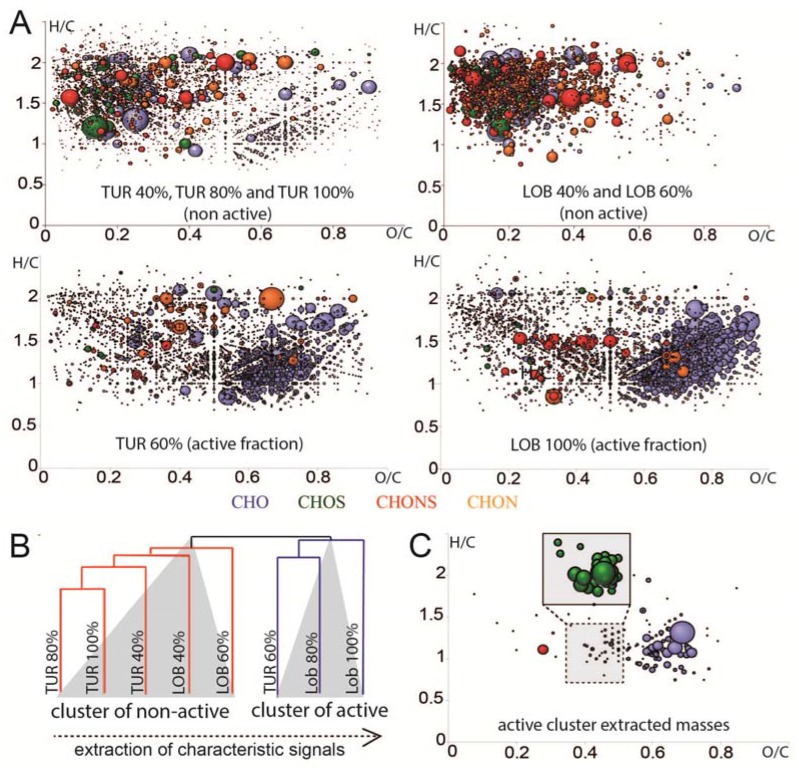
Chemical analysis of HIV-1 RT inhibitory algal fractions of *Turbinaria turbinate* (TUR) and *Lobophora variegata* (LOB) (**A**) Van Krevelen diagrams of non-active algal fractions and active algal fractions based on assigned molecular series (CHO blue, CHON orange, CHONS red, CHOS blue; C: carbon, H: hydrogen, O: oxygen, N: nitrogen, S: sulfur). The illustrated bubble size corresponds to the maximal detected intensity. Enrichment of particularly putative polyphenols is obvious in the active fractions (CHO in H/C 0.6–2 and O/C 0.5–1); (**B**) The mass lists were imported in HCE software for cluster analysis, which revealed a clustering in two groups, active and non-active fractions. In the following, masses exclusively abundant or highly increased in the active fractions are extracted and plotted in a van Krevelen diagram (**C**). Two main groups of compounds are visible (i) polyphenols and (ii) sulfur-containing molecules, which are enlarged and illustrated in the box due to their lower detected intensity.

We analyzed the chemical nature of the HIV-1 RT inhibitory fractions via FT-ICR-MS. Elemental compositions of the detected mass signals were computed via the mass defect based network approach and plotted in van Krevelen diagrams (Figure 5). A comparison of very complex active and non-active fractions for both species points towards an enrichment of compounds within an oxygen-to-carbon (O/C) range of 0.5–0.1 and hydrogen-to-carbon (H/C) range of 0.6–2. This region is usually occupied by oxygen rich polyphenolic compounds, such as anthocyanids or tannins. The assigned molecules contain carbon, hydrogen, and oxygen; their chemical nature being polyphenols or their glycosides is thus reasonable.

The detected masses were afterwards imported in HCE software for cluster analysis, which revealed a close grouping of active and non-active fractions using Euclidian correlation and complete linkage. Using this cluster, masses that were exclusively present or selectively enriched in the active SPE fractions were extracted and plotted in Van Krevelen as well. Four of the filtered masses and proposed elemental compositions (or rather Kegg registered structures) were chemically related to NRTIs in terms of O/C and H/C compositional spaces in the van Krevelen (with 1 hit in the National Institute of Allergy and Infectious Disease database (NIAIDS)), whereas 55 others showed a typical pattern of phenolic compounds. Two of these polyphenolic structures dominated the group by means of their abundance in the sample. These two might therefore be the most interesting for follow up studies. Sixteen of the minor present polyphenols are registered in the NIAIDS database and might contribute to the activity as well. Nineteen compounds belong to a sulfur-containing compound group, all characterized by two sulfur atoms within their molecules. However, except for three, the detected compounds are not registered in any database. The isotope patterns of assigned formulas were simulated and matched the experimental data very well, which verifies the calculated elemental composition.

**Figure 6:**
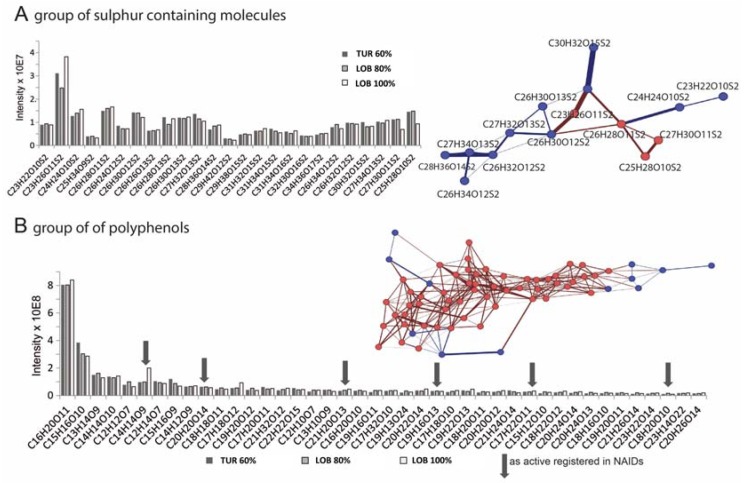
(**A**) Column plot of the detected intensities for sulfur-containing compounds, which are extracted from the cluster analysis separating active and non-active brown algae fractions. This group of compounds clustered very closely together in the mass defected based network, indicating structural similarity and functional variations (right side, blue color indicated exclusive detection in active fractions, red color stands for a detection in active fractions and to a much smaller extent in non-active fractions); (**B**) Column plot for polyphenols with a detected intensity higher than 1 × 10^7^. Two polyphenols are particularly highly present. Matches to the NIAIDS database (which are registered as active) are indicated by an arrow (compare Table 2). The compounds group very closely together in the network (right side, blue color indicates an exclusive detection in active fractions, red color stands for a detection in active fractions and to a much smaller extent in non-active fractions).

The same molecules were selectively illustrated in the networking approach (Figure 6), which uses common biotransformation to interconnect the detected molecules and as a consequence to obtain an impression of whether the molecules of interest might be related to each other, e.g., in terms of a product adduct relationship in metabolic reactions [37,38]. Indeed, the polyphenolic group and the sulfur containing molecules group closely together in this network.

## 3. Discussion

### 3.1. Using HCS as a Broad-Spectrum Technology for Bioprospecting

Cytological profiling of compound-induced perturbations of human cells by High-Content Screening (HCS) has demonstrated its power for the characterization and classification of biological activities of chemical compounds in recent years [29,30,32,34,35]. While most studies that use cytological profiling are focusing on nuclear parameters and, in some cases, the cytoskeleton [32,35], our strategy was to provide a more complete picture of cellular morphology and physiology by including a comprehensive panel of diverse cytological markers [33]. As such, we used HCS as an open-target technology demonstrating a broad assessment of biological activities from samples of unknown composition and a plethora of information on various levels.

### 3.2. Bioprospecting of Marine Algae from the Red Sea Reveals Common Active Fractions and Correlative Trends Across Cellular Markers

Cluster analysis of all cytological profiles from the various algal fractions provided both, a broad overview and detailed insights into the underlying principles of activity. Whereas one third of the tested fractions did not show notable effects on any of the cellular markers, the most activity was found in fractions eluted with higher concentrations of methanol. Higher methanol concentrations favor the elution of non-polar compounds that are usually considered to have more “drug-like” properties, including, e.g., better cell penetration [39,40].

Activity was evenly distributed over all the tested cytological markers highlighting the significance of all markers for the assessment of biological activity as well as the increase of resolution by including a broad panel of cytological markers. While compound-induced toxicity on cancer cells is a widely used readout for activity in natural product screens [24,41,42,43], our study suggests that this uni-dimensional approach tends to miss a majority of activity in biological samples. Although toxicity correlates reasonably with cell cycle interference, it is absent in many fractions where strong effects on markers other than the nucleus were demonstrated. The majority of high-methanol fractions showed activity on multiple cellular markers, while fractions eluted with lower methanol concentrations tended to affect fewer or only single cellular markers. Fractions that influence single markers without affecting other related processes (e.g., activation/inhibition of NFkB or p53 independent of toxicity or cell cycle arrest) could provide interesting starting points in the search for selective activators or inhibitors of specific cellular processes. Thus, cytological profiling can significantly contribute to the development of more targeted therapeutics with reduced side effects.

Notably, the cluster analysis revealed clusters that contained almost exclusively fractions of one species or taxonomic group. This indicates that evolutionary relatedness might lead to a matching set of compounds and could give hints for specific groups of active molecules in these clusters, e.g., tannins or sulfated glycans in the case of brown algae.

### 3.3. HCS Predicts MoAs by Comparative Analysis to Reference Compounds

Interference with the cell cycle of cancer cells is a common therapeutic mechanism that is shared by almost all anti-tumor drugs on the market. Various mechanisms and regulatory pathways have been described that ultimately lead to an arrest of the cell cycle [31,32,44]. In our study, a minor proportion (18%) of fractions were found to have an effect on the cell cycle and most of these showed a clear correlation with toxicity, induction of nuclear features, NFkB activation, and a strong block in the G2/M phase. Cell cycle arrest was found from fractions of one green alga (COD), two red algae (PEY1 and PEY2), as well as from the two species of the brown algal genus *Cystoseira* (CYS1 and CYS2). Cell cycle arrest in G2/M or the induction of apoptosis has been described from a glycoprotein of *Codium decoraticum* [45] as well as a cholesterol derivative from *Codium* sp. [42]. In contrast, two other studies that used methanol extracts of *C. bursa* from Turkey or *C. fragile* from Britain were not able to find any toxicity on mammalian cancer cells [26,43]. Regarding the genus *Cystoseira*, several studies demonstrated cytotoxic effects on cancer cells from *C. sedoides* [24], *C. myrica* [41,46], and *C. tamariscifolia* [43], whereas *C. compressa* and *C. sinuosa* did not show toxic effects on human cancer cells [46,47]. Only very few studies exist on species of the genus *Peyssonnelia*. Two unusual oxylipins from *P. caulifera* showed DNA methyltransferase activity [48,49]. Another study found cytotoxic effects of sterol glycosides from a Fijian *Peyssonnelia* species on human cancer cells [50].

Cytological profiles provide deep insight into the effects that cause an arrest of the cell cycle. By including cytological profiles of reference compounds with known MoAs, the biological targets of test compounds or fractions can be predicted [31,32,33]. Interestingly, most cell cycle-active algal fractions clearly clustered with inhibitors of CDKs or tubulin polymerization. Agents that interfere with tubulin polymerization or lead to a disruption of microtubules are well known to result in a cell cycle arrest in the G2/M phase [51]. Three fractions showed a good concordance with TPI, but differed with regards to actin-related as well as ER and lysosomal features, pointing to an involvement of both actin and tubulin in the arrest of the cell cycle. It is unlikely that the observed effect is a cumulative activity of classical TPI and another compound, as several key features of known TPIs are missing, e.g., effects on ER and lysosomal features. The intermediate cytological phenotype in between TPI and CDK inhibitors of both cell cycle-active *P. squamaria* fractions as well as the unusual arrest of the cell cycle in G0/G1 by the PEY2 fraction (characterized by strong effects on the plasma membrane) clearly demonstrates that algae may constitute a source of new compounds with various interesting mechanisms of cell cycle interference. The intermediate cytological phenotype might reflect the close interplay of tubulin dynamics and CDK activity as demonstrated by the taxol-related upregulation of a CDK inhibitor in human breast cancer cells [52]**,** as well as the finding that activation of CDKs induces important changes of tubulin dynamics. CDK inhibitors such as roscovitine inhibit tubulin phosphorylation and thus have an effect on tubulin formation [53]. Cell cycle inhibition in G0/G1 has been shown to be a promising target in various forms of cancer [54] and was demonstrated to be a result of CDK inhibition by natural products in several cases [55,56,57,58]. Although highly interesting natural products have been isolated from a species of the genus *Peyssonnelia*, it is unlikely that these compounds are responsible for the G0/G1 arrest observed in this study. Peyssonenynes have been shown to inhibit DNA methyltransferase activity [48,49] and this activity is known to result in a cell cycle arrest in the G2/M phase [59,60], which we did not observe.

### 3.4. Successful *In Silico* HIV-1 RT Target Predictions via Profile Comparison to Known Compounds

Marine algae are well known to harbor strong anti-viral activities and several studies demonstrated potent effects on single enzymes or even the full replication of HIV-1 [7,8,9]. The reverse transcriptase (RT), a key enzyme of HIV-1 has been identified as one of the major targets for anti-viral intervention [7]. We were interested to see if cytological profiling is capable of assisting in the identification of algal fractions that harbor potential inhibitors of viral enzymes. In fact, the cytological profiles of three brown algae matched closely to a set of reference nucleosidic HIV-1 RT inhibitors. By using an enzyme-based HIV-1 RT assay, we were able to confirm the inhibitory potential of the selected fractions, thus validating the predicted viral target. Linking a cell-based profile to the activity of an unrelated (viral) single protein target clearly demonstrates the power and great potential of cytological profiling as an open-target technology for the identification of novel activities and the prediction of biological targets or MoAs. Several classes of brown algal natural products have been demonstrated to show inhibitory activity on HIV-1 RT, including phlorotannins, fucans, and diterpenes [7]. Among these, fucans isolated from *L. variegata* were found to have anti-HIV-1 RT activity in vitro [18].

### 3.5. Deep Chemical Profiling of Active Fractions

The combination of resolution, sensitivity, and structural significance makes ICRFT-MS an ideal tool for non-targeted natural product analysis. The wealth of information allows selective filtering (using statistical tools) without biasing the analysis with a priori hypotheses. Ultrahigh resolution is a key parameter, since it leads to the depiction of molecular complexity, accurate assignment of detected masses to elemental formulas (<100 ppb mass error), and annotation of currently unknown compounds [37]. The mass spectra of all analyzed algal extracts allowed a deep insight into their complex composition with approximately 2500 annotated compounds in each fraction. We chose van Krevelen diagrams to visualize this wealth in composition, since it allows for graphically differentiating and separating the classes of compounds. Besides common features, all SPE fractions clearly differed from each other. Comparative data filtering led to a strongly reduced number of compounds, which are presumed to be the active molecules and which will be therefore targeted in follow-up studies. The preparation of biological extracts by SPE provided a ready-to-use starting material due to the reduction of complexity (fractionation according to polarity) and desalting, which is highly required if the samples are of marine origin [31,39,61,62].

Regarding the analyzed cell cycle active fractions, six highly present molecules were characteristic for the active CYS1 fraction. All of these are not currently listed in any database, but are presumably chemically related to each other. Any established cell cycle interfering compounds were absent in the analyzed fractions.

Cluster analysis of anti-HIV-1 RT active and non-active fractions revealed compositional similarities of active fractions. Molecules, which are descriptive for active fractions could be categorized according to their chemical properties into polyphenols, sulfur-containing compounds, and a third category of a very diverse nature and minor presence in the spectra, but which interestingly closely resembles known NRTIs in the van Krevelen diagrams, which might suggest chemical familiarity. The extracted masses were searched in the NIAIDS database, which holds structural and activity-related information of compounds that have been tested against HIV, HIV enzymes, or opportunistic pathogens. It appears that 16 of the polyphenolic compounds and one compound of the NRTI-related group do have an entry. Six of these masses are listed as inactive, eight as medium active, and three as very active. In particular, the NRTI-related compound (C9H12FN3O4) as well as two putative tannins (C14H14O9 and C20H20O14) are listed with HIV-1 RT as the main target (Table 2). However, these compounds have a minor presence in the spectra compared to the other filtered active mass signals, and we therefore speculate that the registered compounds might have contributed to the activity. However, due to the detected intensities that were several orders of magnitude higher, the not yet described compounds might be the major active compounds. Due to the novelty of the detected compounds, we were unable to purchase these molecules for re-testing in the assay. However, chemical synthesis as well as purification from the complex algal extracts is ongoing.

In summary, our work demonstrates the potential and versatility of cytological profiling for the bioprospecting of unknown biological sources. Our work yielded detailed information on promising activities that might serve as a starting point for future studies on the bioactive potential of selected macroalgal species of the Red Sea.

## 4. Materials and Methods

***Sample Collection****.* Macroalgae were collected during diving at several locations at Al Fahal reef (22°17′40.51′′ N; 38°57′55.13′′ E), Inner Fsar reef (22°14′37.61′′ N; 39°00′28.03′′ E), and Thuwal mangrove area (22°16′52.03′′ N; 39°05′06.40′′ E) in March 2013 (for details see Table 1). Directly after collection, any visible contamination was removed from the algal specimens, thalli were rinsed thoroughly with sterile seawater, and frozen in liquid nitrogen. 

***Sample Fractionation***. Organic extracts were prepared by the addition of 1 mL methanol (Sigma-Aldrich, St. Louis, MO, USA) to 100 mg of powdered algal material. Samples were briefly vortexed and extracted at 4 °C overnight. Subsequently, samples were centrifuged at 13,000 *g* for 30 min to remove particulate material and were then stored at −20 °C. For fractionation, methanol extracts were dried and re-dissolved in purified (Licrosolv, Sigma-Aldrich), acidified water (pH 2.0). The extracts were fractionated using SPE cartridges (BondElut C18, Agilent, Santa Clara, CA, USA). Cartridges were conditioned with 1 mL of methanol, 5 mL purified water, and 1 mL of purified, acidified water. Subsequently, 2 mL of each sample were loaded and eluted with 500 μL of eluent of increasing methanol concentration (20%, 40%, 60%, 80%, and 100%). Eluted fractions were dried, re-dissolved in cell culture medium, and used for HCS.

***Cell Culture***. HeLa cells (parental HeLa cell line, NIH AIDS reagents and reference program) were kept under standard conditions at 37 °C in 5% CO_2_ in Dulbecco’s modified Eagle medium (DMEM containing GlutaMAX-1; Life Technologies, Carlsbad, CA, USA) supplemented with 10% fetal bovine serum (Life Technologies) and 1% antibiotic-antimycotic solution (Life Technologies).

***High-Content Screening and Analysis***. HeLa cells were transferred to 384-well plates at a density of 2000 cells per well in a volume of 25 μL of cell culture medium and kept under standard conditions for 24 h. Cells were treated with 25 μL of re-dissolved fractions in 4 replicates. 24 h after treatment, four different cell-staining protocols (panels) were used to stain for 10 cellular targets. In all cases, the cells were fixed with 4% formaldehyde for 20 min. For permeabilization, blocking, and washing steps, HCS-optimized reagents were used (Cellomics HCS reagents Wash Buffer (WB), Wash Buffer II (WBII), Blocking Buffer (BB) and Permeabilization Buffer (PB), Thermo Fisher Scientific, Waltham, MA, USA). Following the last staining, all plates were washed three times with WB, sealed, and stored at 4 °C until further use. Panel 1: Fixed cells were permeabilized for 15 min and blocked for 15 min. 12.5 μL of the primary staining solution containing 3.6 μL/mL phalloidin-FITC (Sigma Aldrich) and 1.3 μL/mL of beta-tubulin antibody (Thermo Fischer Scientific, Waltham, MA, USA) were added per well for 1 h. After two washing steps with BB, 12.5 μL of the secondary staining solution was added (1:500 GAM-DyLight 550, Thermo Fischer Scientific, in BB) for 1 h. Subsequently, cells were washed 3 times with WB and the nuclei were stained with 0.1 μL/mL of Hoechst33342 (Thermo Fisher Scientific). Panel 2: Cells were incubated with stains for the ER (1 μL/mL, ER-Tracker Blue-White DPX, Life Technologies) and lysosomes (0.2 μL/mL, LysoTracker Red DND-99, Life Technologies) in pre-warmed cell culture medium for 30 min under standard conditions. After fixation, cells were washed twice with WB and incubated with a solution of a labelled wheat germ agglutinin (5 μL/mL, Wheat Germ Agglutinin, Alexa Fluor^®^ 488 Conjugate, Life Technologies) for 10 min. Panel 3: Cells were incubated with a solution of mitochondrial dye (0.17 μL/mL, MitoTracker^®^ Orange CMTMRos, Life Technologies) in cell culture medium for 30 min under standard cell culture conditions. After fixation, cells were permeabilized, washed twice with WB, and incubated with the primary staining solution, including the antibody for NFkB (Thermo Fisher Scientific) for 1 h. After removal of the primary antibody solution, cells were incubated with WBII for 15 min, washed twice with WB, and incubated with the secondary staining solution (1:500 GAR-DyLight 550, Thermo Fischer Scientific, in WB) for one hour. Subsequently, cells were incubated with WBII for 10 min and stained with a solution of Hoechst33342 (0.1 μL/mL, Thermo Fisher Scientific) afterwards for another 10 min. Panel 4: After fixation, cells were permeabilized for 17 min, washed twice with WB, and blocked for 30 min. After removal of BB, cells were incubated with the primary antibody solution (5.5 μL/mL of p53 antibody and 1.5 μL/mL of caspase 9 antibody, both Thermo Fisher Scientific, in blocking buffer) for 1 h. After two washing steps with WBII and one washing step with WB, the secondary staining solution (1:500 GAR-DyLight 488 and 1:500 GAM-DyLight 550, both Thermo Fischer Scientific, in WB) was added for 1 h. After removal of the staining solution, cells were washed once with WBII and stained with Hoechst33342 for 10 min.

For High-Content Analysis, the Cellomics Array Scan VTI (Thermo Fisher Scientific) platform equipped with a 10× objective (Zeiss Plan Neofluar, NA 0.3 was used. Images were analyzed using the Compartmental Analysis Bio Application (Cellomics, Thermo Fisher Scientific). At least 500 valid objects were analyzed per well. Cell cycle analysis and analysis of cell loss were accomplished by using the Cell Cycle Bio Application (Cellomics, Thermo Fisher Scientific) using a minimum of 2000 valid objects.

***Data Analysis***. Raw data from automated image analysis for each cytological feature were related to corresponding values from control wells where the control was set to 1. All cytological features of a given fraction or reference compound were combined to result in a cytological profile. All cytological profiles were subjected to hierarchical clustering using complete linkage clustering with optimized gene leaf order and a Pearson correlation using Multi Experiment Viewer (MeV v4.9, Dana-Farber Cancer Institute, Boston, MA, USA). In order to compare the level of activity of cellular markers, distinct thresholds were applied. A cellular marker was considered as showing activity if at least one cellular feature was exceeding or fell below a defined threshold. These thresholds were as follows: (i) Toxicity: below 70% remaining cells; (ii) Cell Cycle: ±1 standard deviation from the mean; all other cellular markers: ±2 standard deviations from the mean).

***HIV-1 RT Activity***. For the analysis of HIV-1 reverse transcriptase activities, the EnzChek^®^ Reverse Transcriptase Assay Kit (Molecular Probes, Life Technologies) was used according to the manufacturer’s instructions. PicoGreen fluorescence was analyzed in a SpectraMax reader (Molecular Devices, Sunnyvale, CA, USA) using the 480/520 nm filter set.

***Chemical Analysis***. Selected SPE fractions of the brown algae CYS1, TUR, and LOB were analyzed by a 12 Tesla Ion Cyclotron Resonance Fourier Transformation Mass Spectrometer (FT-ICR-MS; SolariX, Bruker, Bremen, Germany) coupled to an Apollo II Electrospray Ionization source (ESI; Bruker, Bremen, Germany) in positive mode. The samples were injected at a flow rate of 120 μL/h. All spectra were acquired with a time domain size of 2 M Word within a mass range of 100–1300 *m*/*z*. For each spectrum, 300 scans were accumulated. All fractions were diluted 1 to 50 with an 80% methanol water mixture and transferred to a Gilson Auto Sampler 223 (Gilson Incorporated, Middleton, Wisconsin, WI, USA). The obtained mass spectra were internally calibrated and aligned. Thereafter, the elemental compositions of abundant compounds were calculated by a mass defected based network approach [38] to be able to describe molecules, which are clearly present but not yet registered in any database (which is the case for most natural products). In the following step, the mass signals, which are characteristic for biologically active SPE fractions, were extracted using Hierarchical Clustering Explorer software (http://www.cs.umd.edu/hcil/hce/). These signals are either exclusively present or tremendously increased in biologically active fractions and are therefore most probably responsible for the observed activity.

## Figures and Tables

**Table 1:** Summary of macroalgal species from Central Red Sea coral reefs and mangrove areas assayed in this study. (AFH: Al Fahal reef, IFS: Inner Fsar reef, inner: landward side of reef, outer: ocean-facing side of reef, ID: NCBI Taxonomy database ID.

Species	Code	Phylum	Order	Family	Taxonomy ID	Location	Depth
***Sargassum ilicifolium***	SAR	Phaeophyceae	Fucales	Sargassaceae	246892	AFH, inner	0.5 m
***Turbinaria turbinata***	TUR	Phaeophyceae	Fucales	Sargassaceae	91044	AFH, inner	0.5 m
***Cystoseira foeniculacea***	CYS1	Phaeophyceae	Fucales	Sargassaceae	590109	AFH, inner	0.5 m
***Cystoseira myrica***	CYS2	Phaeophyceae	Fucales	Sargassaceae	590108	AFH, inner	0.5 m
***Lobophora variegata***	LOB	Phaeophyceae	Dictyotales	Dictyotaceae	157001	IFS, outer	10–20 m
***Peyssonnelia squamaria***	PEY1	Rhodophyta	Gigartinales	Peyssonneliaceae	339596	AFH, outer	10–20 m
***Peyssonnelia capensis***	PEY2	Rhodophyta	Gigartinales	Peyssonneliaceae	367055	AFH, outer	10–20 m
***Hildenbrandia*** **sp.**	HLD	Rhodophyta	Hildenbrandiales	Hildenbrandiaceae		AFH, outer	10–20 m
***Bangia*** **sp.**	BAN	Rhodophyta	Bangiales	Bangiaceae		Mangrove	0.5 m
***Actinotrichia fragilis***	ACT	Rhodophyta	Nemaliales	Galaxauraceae	268562	AFH, outer	10–20 m
***Galaxaura rugosa***	GAL	Rhodophyta	Nemaliales	Galaxauraceae	268570	AFH, outer	10–20 m
***Codium fragile***	COD	Chlorophyta	Bryopsidales	Codiaceae	3133	AFH, inner	8 m

**Table 2:** NIAIDS registered molecular formulas computed via the mass defect based network (mass error <0.1 ppm) and extracted from active fractions using hierarchical clustering. Compare with Figure 6 and note the minor presence of these compounds compared with non-registered putatively active compounds.

Experimental Data				NIAIDS Database		
*m*/*z*	Intensity	S/N	Assigned Molecular Formula	EC50 (Cell)	Target	Compound Class
457.111	1.49 × 10^6^	5.8	C21 H22 O10	20 μg/mL	Rnases H, gp120	Polyphenol, Flavonoids
297.097	1.56 × 10^6^	8.7	C14 H16 O7	45.9 μM		Polyphenol,
211.060	4.17 × 10^6^	45.9	C10 H10 O5	inactive		Polyphenol,
447.127	4.28 × 10^6^	10.9	C20 H24 O10	>46.8 μM		Polyphenol, Cycloalkanes
473.142	5.85 × 10^6^	22.2	C22 H26 O10	inactive		Polyphenol, Biphenyls
367.175	7.59 × 10^6^	49.1	C19 H26 O7	>3.4 μM		Polyphenol,
303.011	7.72 × 10^6^	28.1	C12 H8 O8	inactive		Polyphenol, Cage Compounds
419.095	1.22 × 10^7^	44.9	C18 H20 O10	>2.63 μM		Polyphenol,
425.106	2.94 × 10^7^	75.8	C17 H22 O11	12.4 μM		Polyphenol,
475.049	3.18 × 10^7^	89.6	C19 H16 O13	8 μg/mL cell	IN	Polyphenol, Pyrrolidines
289.032	3.61 × 10^7^	170.8	C12 H10 O7	inactive		Polyphenol, Carboxylic Acid Derivatives
503.080	3.95 × 10^7^	106.2	C21 H20 O13	4 µg/mL		Polyphenol, Flavonoids
499.179	4.49 × 10^7^	120.4	C21 H32 O12	>75.6 μM		Polyphenol,
507.075	6.10 × 10^7^	171.1	C20 H20 O14	50 μM	RT	Polyphenol, Tannins
363.069	9.22 × 10^7^	397.6	C15 H16 O9	inactive		Polyphenol, Benzopyrans
349.053	1.32 × 10^8^	718.3	C14 H14 O9	18 μM	RT	Polyphenol, Tannins
268.070	6.62 × 10^6^	77.3	C9 H12 F N3 O4	>20 μM, 0028 μM	RT	Pyrimidine Nucleosides
